# Reopenable clip over‐the‐line method in endoscopic full‐thickness resection of gastric submucosal tumors: A historical control study

**DOI:** 10.1002/deo2.70067

**Published:** 2025-01-28

**Authors:** Satoki Shichijo, Noriya Uedo, Hitoshi Mori, Yushi Kawakami, Yasuhiro Tani, Hiroyoshi Iwagami, Minoru Kato, Shunsuke Yoshii, Takashi Kanesaka, Koji Higashino, Tomoki Michida, Ryu Ishihara, Naoki Shinno, Hisashi Hara, Yoshitomo Yanagimoto, Kazuyoshi Yamamoto, Takeshi Omori

**Affiliations:** ^1^ Department of Gastrointestinal Oncology Osaka International Cancer Institute Osaka Japan; ^2^ Department of Gastroenterological Surgery Osaka International Cancer Institute Osaka Japan

**Keywords:** abdominal pain, endoscopic full‐thickness resection, endoscopy, gastrointestinal stromal tumors, suture techniques

## Abstract

**Objectives:**

Endoscopic full‐thickness resection for gastric submucosal tumors is gradually gaining popularity, and secure and amenable closure is key to its success. This study aimed to compare the reopenable clip over‐the‐line method with the purse‐string method for defect closure after endoscopic full‐thickness resection for gastric submucosal tumors.

**Methods:**

This historical control trial included 37 consecutive patients with 37 gastric submucosal tumors, who underwent endoscopic full‐thickness resection between January 2021 and July 2024. All lesions were resected en bloc. After excluding three patients who underwent non‐full‐thickness resection, 34 patients were analyzed. Post‐endoscopic full‐thickness resection defects were closed using the purse‐string method (*n* = 18) until 2022 and the reopenable clip over‐the‐line method (*n* = 16) from 2023.

**Results:**

The median (interquartile range) time for defect closure was longer in the reopenable clip over‐the‐line method group of 33 (31–57) min than in the purse‐string method group of 26 (24–35) min (*p* = 0.013). The visual analog scale pain score at the umbilical region was lower (*p* = 0.048) after the reopenable clip over‐the‐line method than after the purse‐string method. In the reopenable clip over‐the‐line method group, post‐procedural abdominal pain was confined to the epigastrium, whereas it extended to the umbilical or left lateral regions in the purse‐string method group. The reopenable clip over‐the‐line method group commenced the diet (*p* = 0.001) and discharged (*p* = 0.024) earlier than the purse‐string method group.

**Conclusions:**

Reopenable clip over‐the‐line method facilitated secure post‐endoscopic full‐thickness resection defect closure, reduced post‐procedural abdominal pain, and shortened the fasting and hospitalization periods after endoscopic full‐thickness resection in gastric submucosal tumors.

## INTRODUCTION

Gastric submucosal tumors (SMTs) are found in 0.3%–0.76% of patients undergoing routine endoscopic examinations,^1^, ^2^ and gastrointestinal stromal tumors (GISTs) account for approximately half of these.^3^, ^4^, ^5^, ^6^ Because GISTs are malignant, the clinical practice guidelines for GIST in Japan (4th edition 2022)^7^ define gastric SMT with histologically proven GIST or lesions with clinical suspicion of GISTs as indications for resection.

Complete surgical excision is the standard method of resection for gastric SMT.^8^, ^9^, ^10^, ^11^, ^12^, ^13^, ^14^ Recently, however, the European Society for Medical Oncology‐ European Network For Rare Adult Solid Cancer‐ Genetic Tumor Risk Syndromes Clinical Practice Guidelines suggested that endoscopic resection is considered for selected cases at sarcoma reference centers with experience in endoscopic surgery.^15^ However, the technique of endoscopic full‐thickness resection (EFTR) is still in its infancy. The development of a secure and amenable closure method for full‐thickness defects is key to standardizing this procedure. Various closure methods are currently utilized^16^, ^17^, ^18^, ^19^, ^20^, ^21^ but in our clinical practice, the purse‐string method has been used for closure of full‐thickness defect after EFTR of gastric SMT. However, we have had difficulty in closing large defects and often experienced post‐procedural localized peritonitis.

Recently, the reopenable clip over‐the‐line method (ROLM) was developed by Nomura et al.^22^ The initial aim of ROLM was to close the post‐endoscopic submucosal dissection (post‐ESD) mucosal wound; however, it is currently applied for post‐EFTR full‐thickness defect closure.^23^, ^24^, ^25^ In this study, we compared the efficacy of ROLM and the conventional purse‐string method for defect closure after EFTR in gastric SMT.

## METHODS

### Study design and settings

This retrospective observational study was conducted at a referral cancer center. The study protocol was approved by the Institutional Review Board (No. 19022–4).

### Pretreatment diagnosis

Conventional gastroscopy, endoscopic ultrasound (EUS), and contrast‐enhanced computed tomography (CT) were performed in all patients. Pretreatment histological diagnosis was conducted using either boring (bite‐on‐bite) biopsy or EUS‐fine needle aspiration biopsy.

### Participants

Consecutive patients (*n* = 37) who underwent EFTR for gastric SMTs as advanced medical care were included in this study. Indications for EFTR in gastric SMTs were: (1) maximum size of 11–30 mm on EUS and CT, (2) non‐epithelial neoplasm in the biopsy, (3) no ulceration, (4) intraluminal growth type (tumor epicenter above the middle line of the muscularis propria on EUS), (5) continuation to the muscle layer on EUS, and (6) histologically proven or clinically suspicious (increasing size, irregular borders, or internal heterogeneity) GIST, or size ≥2 cm.^26^ The indication for each case was confirmed by the multidisciplinary cancer board.

Written informed consent was obtained from all patients for the procedures. The requirement for specific informed consent for study participation was waived because of the retrospective nature of the study and all data used for the analysis were anonymized.

### Endoscopic resection procedure

All procedures were performed under general anesthesia in an operating room. One of the two endoscopists (Satoki Shichijo and Noriya Uedo) with experience of ≥500 upper gastrointestinal ESD and three EFTR cases conducted the procedure, together with two assistants. Therapeutic videoendoscopy (GIF‐H290T or GIF‐2TQ260; Olympus Medical Systems Co., Ltd.) was conducted using a transparent hood (D‐201‐11804 or D‐201‐13404; Olympus), and ESD knives (Flush Knife BT 2.0; Fujifilm Medica Co., Ltd. and ITknife2; Olympus).

**FIGURE 1:**
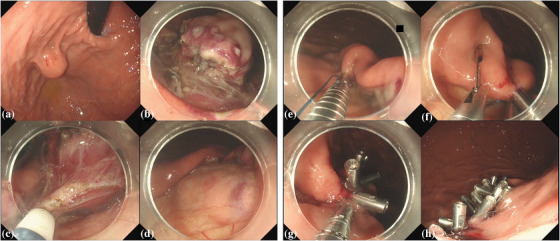
Endoscopic full‐thickness resection and reopenable clip over‐the‐line method. (a) Submucosal tumor at the cardia, intraluminal type size 25 mm. (b) Circumferential exposure of the muscle layer. (c) Intentional full‐thickness resection. (d) Muscle defect after endoscopic resection. (e) A reopenable endoclip tied to the nylon line on the jaw was inserted through the working channel of the endoscope and then applied to the most distal edge of the full‐thickness defect. (f) The full‐thickness layer at the contralateral side of the previous clip was grasped by the next reopenable endoclip, in which the nylon line was threaded through the hole in the jaw. (g) The nylon line was pulled to approximate the two clips. After sufficient approximation of the edges of the full‐thickness defect, the reopenable endoclip was deployed. (h) Complete closure.

The EFTR procedure comprised: (1) circumferential mucosal incision around the tumor, (2) trimming (deepening of the mucosal incision) and submucosal dissection to expose the muscle surface around the tumor, (3) application of clip‐line traction, (4) intentional full‐thickness muscle incision along the mucosal incision line  1 , and (5) post‐EFTR defect closure (Figure 1 and Video 1). The time required for lesion resection was measured from initiation of the mucosal incision until complete detachment of the lesion. The no‐touch EFTR method (mucosal incision 5 mm away from the tumor margin) was adopted from 2023.^25^

### Defect closure

**FIGURE 2:**
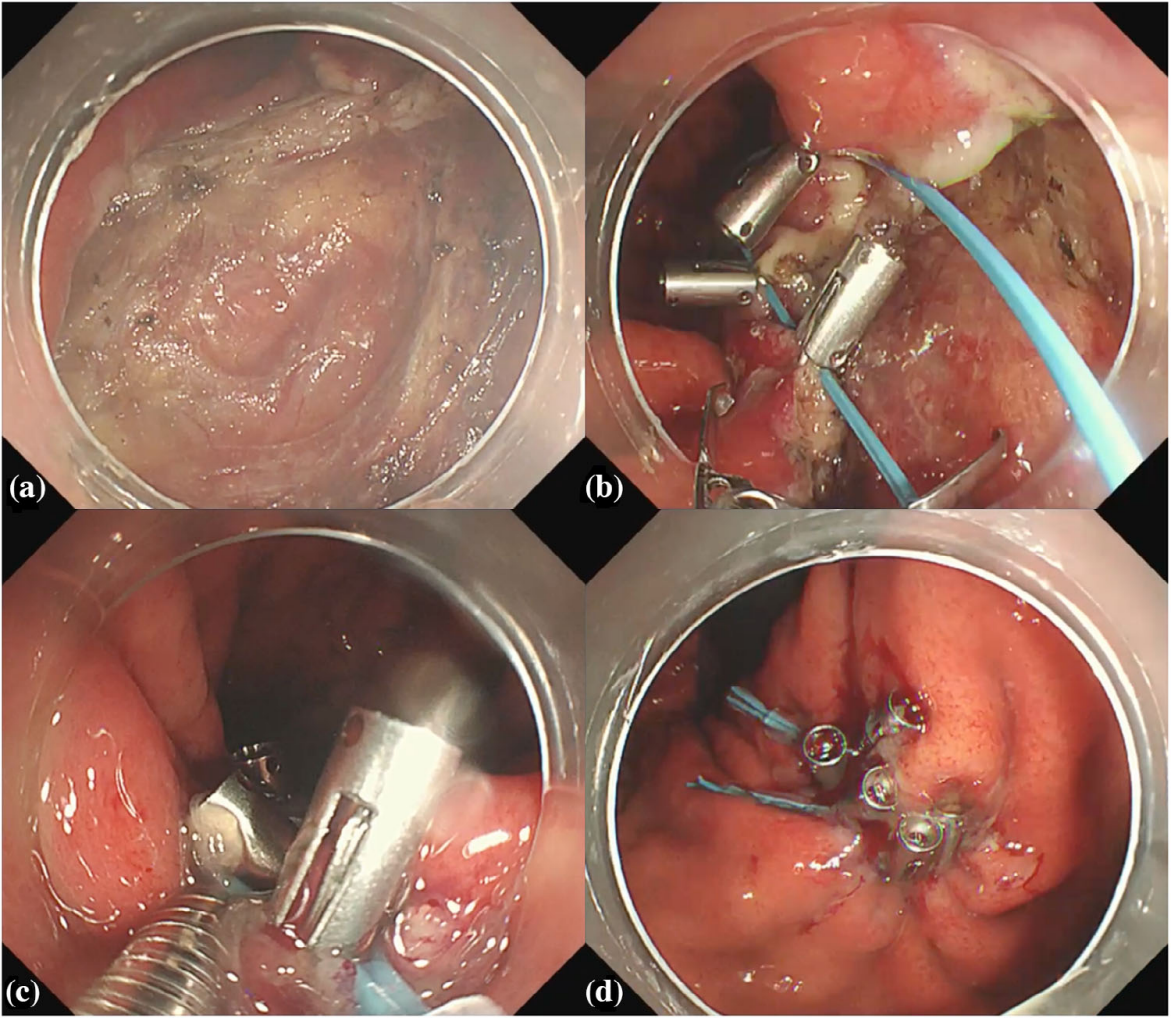
Purse‐string method. (a) Defect after endoscopic full‐thickness resection at the posterior wall of the upper body. (b) An endoloop inserted through the working channel of a double‐channel endoscope was then attached circumferentially to the edge of the mucosa of the full‐thickness defect using endoclips inserted through another working channel of the endoscope. (c) The mucosa was approximated by closing the endoloop when a sheath was used to prevent clips from facing the outside. (d) Complete closure.

To close the post‐EFTR defect, we employed the purse‐string method (*n* = 18) until 2022, and ROLM (*n* = 16) was used from 2023. In the purse‐string method, an endoloop (HX‐400U‐30; Olympus) was inserted through the working channel of a double‐channel endoscope (GIF‐2TQ260) and placed around the defect. The endoloop was then attached circumferentially to the edge of the mucosa of the full‐thickness defect using endoclips inserted through another working channel of the endoscope (SureClip, 11 mm; ROCC‐F‐26‐195‐C; Microtech). The mucosa was approximated by closing the endoloop resulting in complete closure of the defect (Figure 2).

In ROLM,^27^, ^28^ either a single‐ or double‐channel endoscope, reopenable endoclips with a hole in the jaw (SureClip, 16 mm; ROCC‐F‐26‐195‐C; Microtech), and a 3‐0 monofilament nylon suture line (Akiyama Medical Co. Ltd., Tokyo) were used. First, a reopenable endoclip tied to the nylon line on the jaw was inserted through the working channel of the endoscope and then applied to the most distal edge of the full‐thickness defect. Both the mucosa and muscle were grasped using the reopenable endoclip. Subsequently, the end of the line emerging from the accessory channel of the endoscope was threaded through the hole in the jaw of the reopenable endoclip, and the reopenable endoclip was ropewayed through the working channel. The mucosa and muscle adjacent to the first clip were grasped by the reopenable endoclip, placing the jaw with the threaded nylon line inside, and the nylon line was pulled to approximate the two clips. After sufficient approximation of the edges of the full‐thickness defect, the reopenable endoclip was deployed. The same maneuvers were repeated on the contralateral side of the full‐thickness defect until the defect was completely closed. Finally, the line was cut 1 cm away from the last re‐openable endoclip using scissors forceps (FS‐3L‐1; Olympus; Figure 1 and Video 1).

**VIDEO 1:** No‐touch endoscopic full‐thickness resection and reopenable clip over‐the‐line method for defect closure.

### Postoperative management

Antibiotics were administered 30 min before and every 3 hours during EFTR and continued based on the clinical course. A proton‐pump inhibitor was administered from the day of treatment until postoperative day (POD) 28. A nasogastric tube was inserted at the end of the procedure. On POD 1, blood tests and physical examinations were conducted to assess intra‐abdominal inflammation. Abdominal pain was rated using a visual analog scale (VAS) from 1 to 10 at four abdominal locations (epigastric, umbilical, and left and right lateral). If there was no or low‐grade (VAS ≤ 2) abdominal pain and no fever ≥38°C, oral hydration was initiated, and a liquid diet was commenced from POD 2. If abdominal pain was rated > 2, the patient remained nil per os with a nasogastric tube. After an improvement in the abdominal pain, oral hydration was initiated, and a liquid diet was commenced on the subsequent day following the initiation of oral rehydration. Routine CT or endoscopy was not performed. The diet was gradually changed to soft meals, and with no increase in abdominal pain and fever, the patient was discharged after 4 days of resuming the diet.

### Histological analysis

After fixation with formalin, the specimens were sectioned at 3‐mm intervals. For GIST, the tumor risk was classified based on the modified Fletcher classification.^29^ Histologically complete resection (R0) was defined as a clear histological margin of the entire tumor in all sections, with histologically non‐complete resection (R1) defined as obvious histological damage to the tumor margin, and RX as an unclear histological margin.

### Measured outcomes

Outcome variables were time to full‐thickness defect closure, VAS score for abdominal pain on POD 1, white blood cell (WBC) count, neutrophil count, C‐reactive protein (CRP) level on POD 1, days of fasting, admission after EFTR, and adverse events within 30 days according to the Clavien–Dindo classification. The time for full‐thickness defect closure was measured from the end of tumor resection until the end of closure.^26^

### Statistical analysis

Statistical analyses were performed for all eligible cases. In the endpoint analyses, proportions and 95% confidence intervals were calculated for categorical data. Continuous data are presented as median (interquartile range). Categorical variables were compared using chi‐squared or Fisher's exact probability test and continuous variables using the Mann–Whitney U‐test. *P* < 0.05 was considered statistically significant. All statistical analyses were performed using JMP software (version 17; SAS Institute).

## RESULTS

**FIGURE 3:**
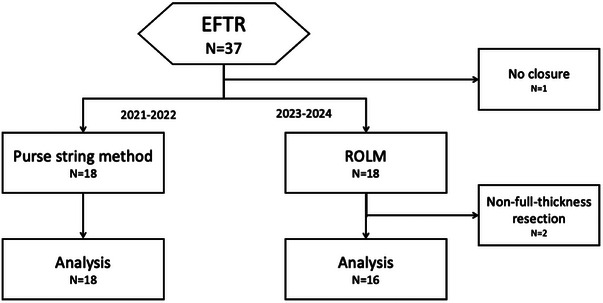
Participant flow.

A total of 37 patients with 37 gastric SMTs underwent EFTR between January 2021 and July 2024. Three patients with lesions confined to the submucosa or inner circumferential muscle were excluded because resection did not result in full‐thickness resection (Figure 3).

Patient background characteristics are presented in Table 1. No significant differences were identified regarding the background demographics of each group, except for the pretreatment histological diagnosis and indication for resection. Endoscopic resection was completed in all patients without intraprocedural adverse events. Abdominal paracentesis for pneumoperitoneum was required in six patients in the purse‐string method group and in four in the ROLM group; lesion resection times were similar in both groups: 57 (41–85) vs. 52 (40–94) min. En‐bloc resection was achieved in all cases. No instances of surgical conversion were reported.

**TABLE 1:** Clinicodemographic characteristics of participants.

	Purse‐string method (*N* = 18)	ROLM (*N* = 16)	*p*‐value
Period	2021–2022	2023–2024	
Age (years)^‡^	54 (46–65)	57 (47–74)	0.387
Sex, *n* (%)			
Male	9 (50)	8 (50)	1.000
Female	9 (50)	8 (50)	
Comorbidities, *n* (%)			
None	7 (39)	8 (50)	
DM	5 (28)	5 (31)	
Hypertension	2 (11)	2 (13)	
Hyperlipidemia	1 (5.5)	2 (13)	
Others	6 (33)	0	
Endoscopic lesion size (mm)^‡^	18 (16–20)	16 (14–25)	0.972
Longitudinal lesion location, *n* (%)			
Upper third	7 (38)	11 (69)	0.188
Middle third	8 (44)	3 (19)	
Lower third	3 (17)	2 (13)	
Circumferential lesion location, *n* (%)			
Anterior wall	6 (33)	3 (33)	0.058
Greater curvature	3 (19)	3 (17)	
Lesser curvature	6 (33)	1 (6.3)	
Posterior wall	3 (19)	9 (56)	
Growth type, *n* (%)			
Intraluminal	18 (100)	16 (100)	1.000
Pretreatment histological diagnosis, *n* (%)			
GIST	10 (56)	2 (13)	0.017
Non‐tumor	6 (33)	13 (81)	
Leiomyoma	2 (11)	1 (6)	
Indication, *n* (%)			
Histologically proven GIST	10 (56)	2 (13)	0.024
Increasing size	5 (28)	11 (69)	
Size ≥2 cm	3 (17)	3 (19)	
Full thickness resection, *n* (%)			
Yes	18 (100)	16 (100)	1.000
No	0	0	
Abdominal paracentesis, *n* (%)			
Yes	6 (33)	4 (25)	0.715
No	12 (67)	12 (75)	
Time for lesion resection (min)^‡^	57 (41–85)	52 (40–94)	0.717
En bloc resection, *n* (%)	18 (100)	16 (100)	

Abbreviations: DM, diabetes mellitus; GIST, gastrointestinal stromal tumors; ROLM, reopenable clip over‐the‐line.

^‡^ Median (interquartile range).

### Defect closure time

The time to defect closure was 26 (24–35) and 33 (31–57) min in the purse‐string method and ROLM groups, respectively (*p* = 0.013, Table 2).

**TABLE 2:** Defect closure and post‐procedural outcome data.

	Purse‐string method (*N* = 18)	ROLM (*N* = 16)	*p*‐value
Time for defect closure (min)^‡^	26 (24–35)	33 (31–57)	0.013
Number of endoclips used	9 (7–10.25)	16.5 (14–21)	<0.001
Completion of closure procedure, Yes; *n* (%)	18 (100)	16 (100)	1.000
Days from EFTR to resume diet^‡^			
2	0	7	0.001
3	12	8	
≥4	6	1	
Days from EFTR to discharge^‡^			
≤ 6	1	7	0.024
7	10	5	
≥ 8	7	4	
Adverse events,*n*			
Delayed bleeding	0	0	1.000
Delayed perforation	1 (Grade IIIa)	0	

Abbreviations: EFTR, endoscopic full‐thickness resection; ROLM, reopenable clip over‐the‐line method.

^‡^ Median (interquartile range).

**TABLE 3:** Histological data.

	Purse‐string method (*N* = 18)	ROLM (*N* = 16)	*p*‐value
Resected specimen size (mm)^‡^	25.5 (21.5–32)	33 (20–32)	0.054
Histological tumor size (mm)^‡^	20.5 (17.75–25)	20 (16.5–30.25)	0.849
Histological diagnosis, *n* (%)			
GIST	12 (67)	8 (50)	0.377
Leiomyoma	3 (17)	6 (38)	
Schwannoma	2 (11)	2 (13)	
Ectopic pancreas	1 (5.6)	0	
Histological complete resection, *n* (%)			
R0	12 (67)	15 (94)	0.102
R1	1 (5.6)	0	
RX	5 (28)	1 (5.6)	
Risk classification of GIST			
High	2 (17)	0	0.243
Intermediate	0	1 (13)	
Low	3 (25)	3 (38)	
Very low	7 (58)	4 (50)	

Abbreviations: GIST, gastrointestinal stromal tumors; ROLM, reopenable clip over‐the‐line.

^‡^ Mean ± standard deviation (range).

### Number of devices used for defect closure

The number of endoclips used was 9 (7–10.25) and 16.5 (14–21) in the purse‐string method and ROLM groups, respectively (*p* < 0.001). In the purse‐string method group, 1 (1–2) endoloop was used per patient. In one early case in the ROLM group, one endoloop with two clips was added to strengthen the closure of the defect edge, whereas two over‐the‐scope clips were used in a case of the purse‐string group, in which closure was difficult because of the thick wall in the antrum.

### Post‐procedural outcomes

**FIGURE 4:**
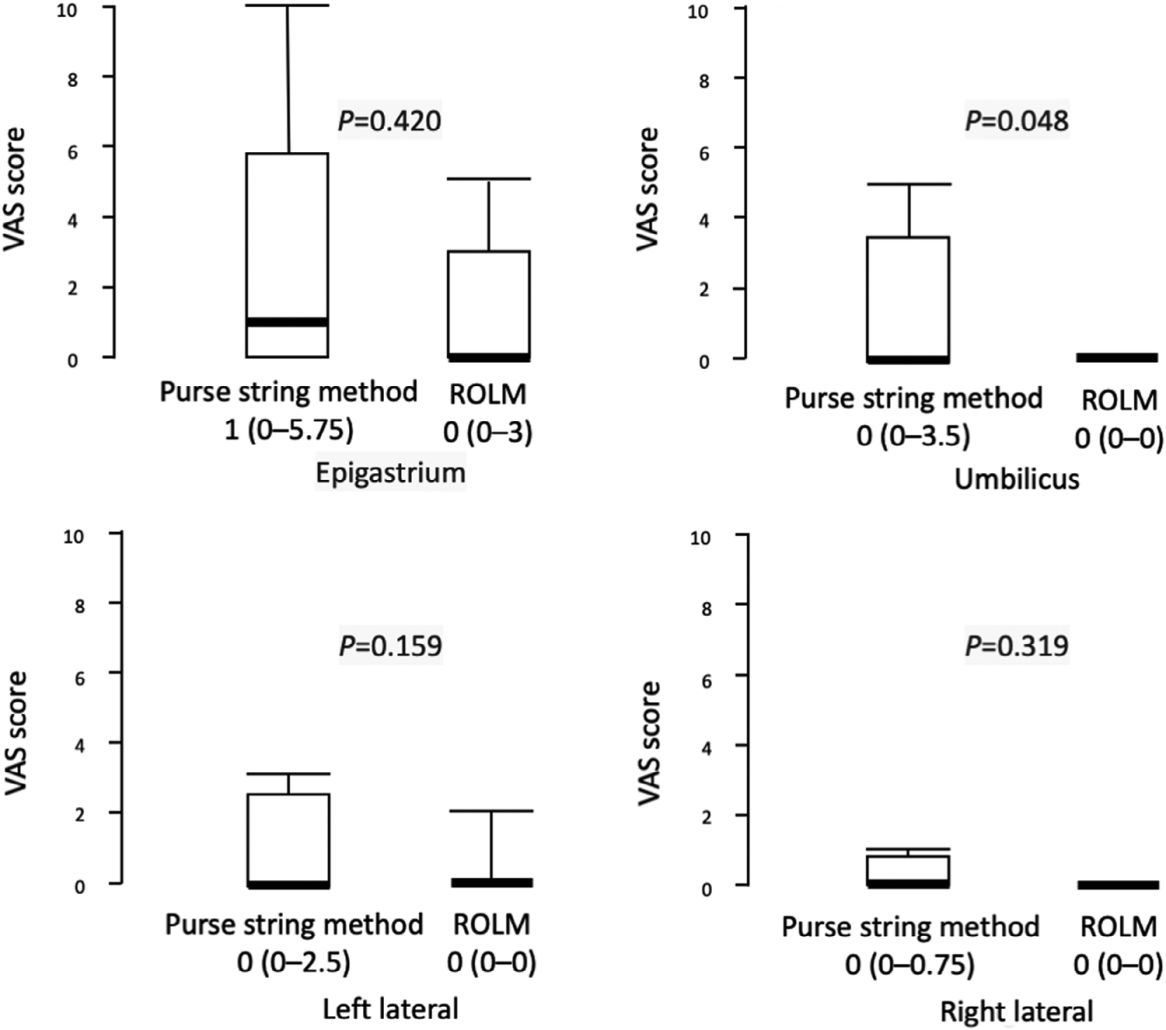
Visual analog scale score for abdominal pain on postoperative day 1.

The VAS scores in the epigastric, umbilical, and left and right lateral abdominal regions for both methods are summarized in Figure 4. Two patients who underwent the purse‐string method did not have their VAS scores assessed on POD 1. The VAS score for the umbilical region in the ROLM group was significantly lower (*p* = 0.048) than that in the purse‐string group. In the ROLM group, post‐procedural abdominal pain was almost exclusively confined to the epigastrium, whereas it extended to the umbilical or left lateral regions in the purse‐string method group.

WBC counts, neutrophil counts, and CRP value in the purse‐string method and the ROLM were 11360 (9297–13,552) versus 11145 (8222–14,680)/mm^3^ (*p* = 0.973); 9895 (7432–11,592) versus 8575 (6270–12,075)/mm^3^ (*p* = 0.654); and 3.32 (0.95–4.8) versus 2.21 (1.44–4.29) mg/L (*p* = 0.730), respectively.

### Fasting and hospitalization periods

The ROLM group (POD 2 in seven patients, POD 3 in eight, and ≥POD 4 in one) started on the diet earlier than the purse‐string method group (POD 3 in 12 patients and ≥POD 4 in six; *p* = 0.001). Patients in the ROLM group (≤POD 6 in seven patients, POD 7 in five, and ≥POD 8 in four) were discharged earlier than those in the purse‐string method group (≤POD 6 in one patient, POD 7 in 10, and ≥POD 8 in seven; *p* = 0.024, Table 2).

### Adverse events

One patient in the purse‐string method group developed peritoneal irritation and CT showed increased free air in the abdominal cavity on POD 1. Emergency endoscopy found a small hole between the approximated mucosa, which was managed by a polyglycolic acid sheet insertion and fibrin glue application (Clavien–Dindo Grade IIIa).^26^

### Histological complete resection

R0, R1, and RX were reported in 12, one, and five patients, respectively, in the purse‐string method group and 15, none, and one, respectively, in the ROLM group (Table 3).

## DISCUSSION

This single‐center retrospective observational study indicated that ROLM resulted in less post‐procedural abdominal pain and reduced the fasting and hospitalization periods after EFTR compared with the purse‐string method.

The purse‐string method, utilizing an endoloop and endoclips, is one of the most common methods for closing post‐EFTR defects in gastric SMT. In the purse‐string method, when the proper muscle is grasped by endoclips, it may move outwards against the gastric wall during endoloop closure. Consequently, the mucosa and submucosa above the muscle are typically grasped and approximated together using an endoloop. In the ROLM, the nylon line that threaded the clip jaw was pulled towards the lumen each time after grasping the tissue; therefore, the muscle layer could be grasped together with the mucosa and submucosa. Strong grasping and approximation forces are thereby provided. The ROLM can also grasp the peritoneum outside the gastric wall,^28^ enabling the peritoneum to be patched.

Laboratory data on inflammation and VAS scores for abdominal pain revealed a lower tendency in the ROLM group, suggesting effective defect closure. As the purse‐string method only approximates the mucosa and submucosa, we assume that a gap may have formed between the endoclips, resulting in minor leakage of gastric content. One patient developed delayed perforation due to insufficient closure of the mucosa. However, the ROLM enables secure full‐thickness closure including the muscle layer, and shorter fasting and hospitalization periods were observed. Initially, we set the day to resume diet as POD 3. However, after the implementation of the ROLM, many patients were able to resume diet from POD 2 and relieved of nasogastric tube discomfort and abdominal pain. Although the ROLM increases the number of endoclips and prolongs procedure time, the reduction in patient discomfort, potential risk of adverse events, and duration of fasting and hospitalization could compensate for these problems.

With the purse‐string method, the closure of large defects during a single procedure is difficult because the size of the defect closure is limited by the size of the endoloop. However, in ROLM, theoretically, no limit exists to the size of the defects that can be closed. Therefore, ROLM enables the implementation of the concept of no‐touch EFTR,^25^, ^30^ in which the lesion is resected together with the surrounding tissue. This results in a higher R0 rate in the ROLM group (94%) than in the purse‐string group (67%), demonstrating the therapeutic efficacy of EFTR for GIST being comparable to that of surgical resection.

In Western countries, the OverStitch endoscopic suturing system^20^ is available. It enables robust suturing by threading the entire gastric wall. In this study, the full‐thickness defect could be closed with commonly used endoclips, endoloops, and a nylon thread. In Japan, one SureClip costs 3500 yen, endoloop 5204 yen, and nylon thread 228 yen, for a total cost of approximately 40,600 yen for the purse‐string method, and 67,000 yen for the ROLM. The purse‐string method and the ROLM could be useful alternatives in countries where access to advanced instruments, such as the OverStitch, is difficult. Additionally, unlike suturing methods that require large working spaces, they can be used to close defects in confined spaces because they only involve maneuvering to grasp the edges of the defects using an endoclip. This characteristic is especially useful when performing procedures where the lumen has collapsed after a full‐thickness resection.

The time required for closure was significantly longer with the ROLM. The main reason for the long procedure time in the ROLM was that each time the clip that grasped the defect edge had to be approximated to the previous clip by pulling the line. However, individual maneuvers are simple and have a higher success rate than the final step of the purse‐string method, in which all clips must be directed toward the gastric lumen. Therefore, this is less stressful for the operator. In one case, the duration of ROLM was prolonged (220 min), in which a 4‐0 nylon line was used because a 3‐0 nylon line was not available, which caused the thread closure to loosen preventing proper closure. However, this issue was resolved when the nylon line was changed to a thicker 3‐0 nylon suture.

This study has some limitations. First, as this was a historically controlled study, improvement in endoscopists' skills might have influenced the outcomes. However, in the purse‐string group, the data were derived from the practice of operators familiar with the technique. In contrast, the ROLM group included data from the first case. Despite such a short learning period, the ROLM showed better closure outcomes even in cases with a large resected specimen size. Second, the procedures were performed by experts at a single center, and their validity was not determined. The steps of the ROLM were initially difficult to understand, and one of the operators visited the developer of the method. Before the implementation of the ROLM to EFTR, we performed the ROLM in several cases of ESD defect closure. Once the basic principles and operating maneuvers were understood, the learning curve was short and the procedure was well replicated as it was a repetition of simple operations.

In conclusion, ROLM facilitated the secure closure of post‐EFTR defects, reduced post‐procedural abdominal pain, and shortened the fasting and hospitalization period after EFTR for gastric SMT. It warrants further validation in wider clinical settings.

## CONFLICT OF INTEREST STATEMENT

Satoki Shichijo received honoraria for lectures from Fujifilm, Boston Scientific Japan, EA Pharma, AstraZeneca, Daiichi‐Sankyo Co., Ltd., AI Medical Service, Zeria Pharmaceutical Co., Ltd., and Janssen Pharmaceutical Co., Ltd.

Noriya Uedo received honoraria for lectures from Olympus Co. Ltd., Fujifilm Co. Ltd., Boston Scientific Japan, Daiichi‐Sankyo Co. Ltd., Takeda Pharmaceutical Co. Ltd., EA Pharma Co. Ltd., Otsuka Pharmaceutical Co. Ltd., AstraZeneca Co., Ltd., 3‐D Matrix Co. Ltd., AI Medical Service, MC Medical Inc., and Miyarisan Pharmaceutical Co. Ltd.

Takashi Kanesaka received honoraria for lectures form Olympus Co. Ltd., AstraZeneca Co. Ltd., and AI Medical Service.

Ryu Ishihara received honoraria for lectures from Olympus Co., Ltd, Fujifilm Co., Ltd, Daiichi‐Sankyo Co. Ltd, Miyarisan Pharmaceutical Co., Ltd, AI Medical Service, AstraZeneca Co. Ltd., MSD Co. Ltd., and Ono Parmaceutical Co. Ltd.

## ETHICS STATEMENT

The study protocol was approved by the Institutional Review Board (No. 19022–4).

## PATIENT CONSENT STATEMENT

N/A

## CLINICAL TRIAL REGISTRATION

Registry and the Registration number of the study: N/A
